# Quality of Life and Sleep in Patients with Pituitary Adenoma in Relation to Tumor Type and Compression of the Optic Chiasm

**DOI:** 10.3390/jcm10091879

**Published:** 2021-04-26

**Authors:** Karol Piotr Sagan, Elżbieta Andrysiak-Mamos, Ernest Tyburski, Leszek Michał Sagan, Anhelli Syrenicz

**Affiliations:** 1 Department of Endocrinology, Metabolic and Internal Diseases, Pomeranian Medical University, 70-204 Szczecin, Poland; elamamos@tlen.pl (E.A.-M.); anhelli@asymed.ifg.pl (A.S.); 2 Institute of Psychology, SWPS University of Social Sciences and Humanities, 03-815 Poznan, Poland; etyburski@swps.edu.pl; 3 Department of Neurosurgery, Pomeranian Medical University, 70-204 Szczecin, Poland; leszekm.sagan@gmail.com

**Keywords:** pituitary adenoma, quality of life, quality of sleep, Cushing’s syndrome, acromegaly, non-functioning pituitary adenoma

## Abstract

Objective: To determine the effect of transsphenoidal surgery on quality of life and sleep in patients with pituitary adenomas depending on tumor type and compression of the optic chiasm. Methods: In this prospective study, patients with pituitary adenomas who were scheduled for transsphenoidal surgery completed the Short Form 36 Questionnaire, Pittsburgh Sleep Quality Index, and Epworth Sleepiness Scale preoperatively and 7.5 (±1.5) months after surgery. Patients were analyzed based on tumor type and compression of the optic chiasm. Results: Significant improvements with large effect sizes were seen for patients with Cushing’s disease in general health (Z = −2.37; *p* = 0.018), vitality (Z = −2.05; *p* = 0.041), and mental health (Z = −2.06; *p* = 0.040). A significant deterioration with large effect size occurred in physical functioning (Z = −2.02; *p* = 0.043) in patients with acromegaly. A significant improvement with medium effect size was seen in subjective sleep quality, (Z = −2.24; *p* = 0.025), sleep duration (Z = −2.11; *p* = 0.035), and habitual sleep efficiency (Z = −2.26; *p* = 0.024) after decompression of the optic chiasm. Multiple significant correlations were observed between sleep parameters and Short Form 36 subscales before and after treatment. Conclusions: Changes in quality of life during the follow-up period depend on tumor type. Circadian rhythm disturbances may resolve promptly after decompression of the optic chiasm. Quality of life in pituitary adenoma patients is associated with quality of sleep in many dimensions, thus implying that developing strategies to improve sleep quality could increase overall well-being and everyday functioning in pituitary adenoma patients.

## 1. Introduction

Pituitary adenomas (PAs) account for 10 to 15% of all brain neoplasms [1]. Due to the specific location of these tumors, their mass effect often leads to pituitary insufficiency, headaches, visual field defects, and disturbed circadian rhythms, with varying outcomes after transsphenoidal surgery (TSS). Additionally, 46 to 86% of PAs are hormonally active [2], causing the development of additional long-term and serious health complications. Thus, given the good survival rates, PAs are especially associated with decreased quality of life (QoL), which may remain unsatisfactory despite successful treatment [3,4,5]. Therefore, from a healthcare point of view, as well as from a case-oriented perspective, assessing the quality of life of PA patients is an important step in developing new treatment strategies for long-term patient care. There has been research in recent years on the QoL of patients treated for PA. Some studies indicate a significant improvement in QoL after TSS, especially for non-functioning pituitary adenomas (NFPAs; [6]), while other reports have found persistently reduced QoL after resection of functional pituitary adenomas (FPA) and NFPAs [7,8]. Importantly, QoL does not only depend on the biological effects of the disease, but is also influenced by other factors, including physical, psychological, and social problems [9]. Studies on the QoL of PA patients from Central Europe are lacking and cannot be extrapolated from other regions due to specific socio-economic and cultural factors.

One of the aspects of care commonly neglected in PA patients is quality of sleep, which affects many aspects of QoL [10]. Patients treated for PA suffer from various sleep disturbances, with many possible causal mechanisms, including pituitary insufficiency, hormone excess, or psychological, behavioral, or environmental factors [11]. Compression of the anatomical structures that form the circadian pacemaker may be the leading cause of disruption of circadian rhythms in patients with adenomas with suprasellar extension [12]. However, to date, there has been no evidence of the reversibility of sleep disorders caused by this pressure effect.

The aim of our study was to determine the effect of TSS on QoL and sleep in PA patients using the Short Form 36 Survey version 2 (SF-36v2), Pittsburgh Sleep Quality Index (PSQI), and Epworth Sleepiness Scale (ESS). We also assessed the relationship between subjective sleep parameters and QoL dimensions before and after tumor resection.

## 2. Materials and Methods

### 2.1. Subjects and Study Design

We prospectively collected data from patients who were qualified for microscopic TSS due to suspected PAs in the period from December 2016 to January 2020 at our hospital. The indication for surgery were FPA or non-functioning macroadenoma with compression of the optic chiasm or symptomatic parasellar extension. The final decision regarding tumor resection was made by a multidisciplinary team (endocrinologist, neurosurgeon, radiologist). Patients with a history of prior pituitary gland surgery were excluded from the study. None of the patients received radiotherapy during the follow-up period.

**Figure 1:**
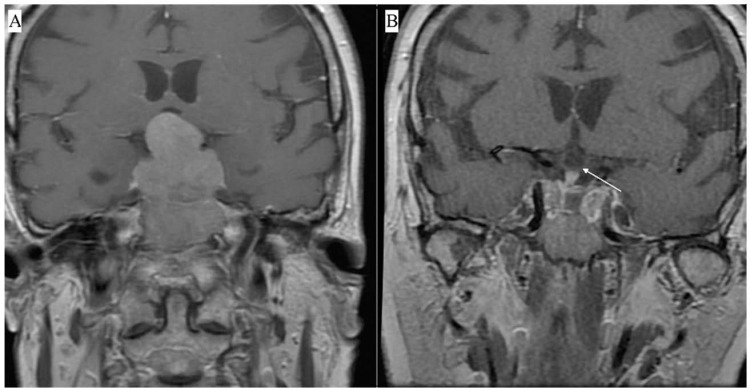
Contrast-enhanced MRI showing a macroadenoma penetrating into the suprasellar region and compressing the optic chiasm. Preoperative T1—weighted contrast-enhanced coronal view (**A**), postoperative T1—weighted contrast-enhanced coronal view. The white arrow indicates the optic chiasm visible after tumor resection (**B**).

Patients were asked to complete the Polish versions of three questionnaires (SF-36v2, PSQI, and ESS) before surgery and 7.5 months (±1.5 months) after PA resection. The questionnaires were completed using pencil and paper. The study was approved by the local ethics committee. Licenses for the questionnaires were obtained from the appropriate institutions. Of 55 consecutive patients scheduled for TSS due to primary radiological and endocrinological diagnosis of a pituitary adenoma, consent was obtained from 43 individuals. All TSSs were performed by a single experienced neurosurgeon. Patients received endocrinological and ophthalmological care before and after the operation. Magnetic resonance imaging (MRI) was performed before and within 9 months after surgery. Figure 1 shows an exemplary MRI of the parasellar region in a patient with pituitary macroadenoma.

Diagnoses of Cushing’s disease and acromegaly were based on the guidelines of the European Society of Endocrinology [13,14]. As inferior petrous sinus sampling was not available at our center at the time of the study, the pituitary source of adrenocorticotropic hormone in Cushing’s disease patients presenting with microadenomas was confirmed by the high dose dexamethasone suppression test of cortisol secretion and/or the corticotropin-releasing hormone stimulation test. Cushing’s disease remission was defined as morning serum cortisol values <5 µg/dL within 7 days of TSS or glucocorticoid dependence assessed by biochemical and clinical evidence. Acromegaly remission was defined as human growth hormone (hGH) <1 µg/L at baseline and low/normal IGF-1 or suppression of hGH <0.4 µg/L in the oral glucose tolerance test (OGTT) and low/normal IGF-1 matched for age and sex. In line with the recommendations of the Polish Society of Endocrinology [15], all acromegaly patients received somatostatin analogues (SSA) prior to TSS. Consequently, three of eight acromegaly patients had documented normal IGF-1 levels before TSS.

Eight patients were excluded from the study due to a histopathological diagnosis other than PA. Four patients with NFPA were lost during follow-up: 3 patients did not return questionnaires and 1 patient with a giant PA died in the postoperative period due to an intracranial hemorrhage. Additionally, 2 patients were excluded during the study: 1 patient was excluded due to inconsistent responses and suspicion of dementia and 1 patient was excluded due to depression, non-compliance, and hydrocortisone withdrawal. All FPA patients completed the study. The results of 29 individuals were included in the statistical analysis. The baseline characteristics of the study group are presented in Table 1.

We assessed the change in QoL and quality of sleep after TSS with the use of validated questionnaires. Patients were grouped by tumor type based on endocrinological diagnosis and according to the presence of optic chiasm compression (POCC) at baseline. Optic chiasm compression has been defined on MRI as displacement of at least the inferior margin of the optic chiasm. We also assessed the correlations between sleep parameters and QoL before and after surgery.

### 2.2. Questionnaires

#### 2.2.1. Short Form 36 Survey Version 2

The Short Form 36 Questionnaire measures 8 domains of health-related QoL [16]: physical functioning (PF), role participation with physical health problems (role-physical; RF), bodily pain (BP), general health (GH), vitality (VT), social functioning (SF), role participation with emotional health problems (role-emotional; RE), and mental health (MH). All health domain scales are scored 0 to 100, with higher scores indicating better health. Three scales (PF, RP, and BP) correlate the most with the physical component. The mental component correlates the most with the MH, RE, and SF scales. Vitality, GH, and SF are correlated with both components.

#### 2.2.2. Epworth Sleepiness Scale

The Epworth Sleepiness Scale is a simple and validated tool developed for clinicians and researchers to measure daytime sleepiness [17]. The total score ranges from 0 to 24, with higher scores indicating greater daytime sleepiness.

#### 2.2.3. Pittsburgh Sleep Quality Index

The Pittsburgh Sleep Quality Index assesses sleep disturbances that affect sleep quality [18]. Scale items generate the seven following component scores: C1—Subjective Sleep Quality, C2—Sleep Latency, C3—Sleep Duration, C4—Habitual Sleep Efficiency, C5—Sleep Disturbances, C6—Use of Sleeping Medication, and C7—Daytime Dysfunction. All components sum to a total score ranging from 0 to 21.

### 2.3. Statistical Analysis

Statistical analysis was done using the IBM SPSS 25 Statistical package. Variables were presented as means. The normalities of the distributions of continuous variables were tested with the Shapiro-Wilk test. Due to the small size of the groups, nonparametric statistics were used. The *Z* Wilcoxon signed-rank test was conducted to evaluate the change in the clinical status of patients between pre- and post-treatment. To determine the effect size between pre- and post-treatment, the *r* rank-biserial correlation method was used: *r* = *Z/*√2*n* [19]. Spearman’s *R* was used to assess the strength of the identified correlations between continuous variables separately, pre- and post-treatment.

## 3. Results

### 3.1. Surgical Outcomes

Of the 29 patients who completed the follow-up, total tumor resection was achieved in 22 patients (76%). Five patients (17%) had a subtotal resection. In two patients (7%), the extent of the resection was uncertain due to discrepancies between the neurosurgeon’s and radiologist’s MRI evaluations. Sixteen patients (94%) with FPAs were fully biochemically and clinically cured of their endocrinopathy. One patient had an indeterminate outcome. This was a patient with acromegaly in whom postsurgical IGF-1 was within the normal range and baseline hGH was 1.36 µg/L. The patient had a history of a mini-gastric bypass and, due to hypoglycemia in OGTT, it was not possible to assess suppression of hGH in this individual. Post-surgical hypopituitarism of at least one hormonal axis occurred in 5/12 (42%) NFPA patients and in 12/17 (71%) FPA patients. Five patients (17%) developed post-surgical diabetes insipidus. Other complications included one case of cerebrospinal fluid leak and one case of pulmonary embolism in the same patient.

### 3.2. Pre- and Post-Treatment Comparison of Quality of Sleep

**Figure 2:**
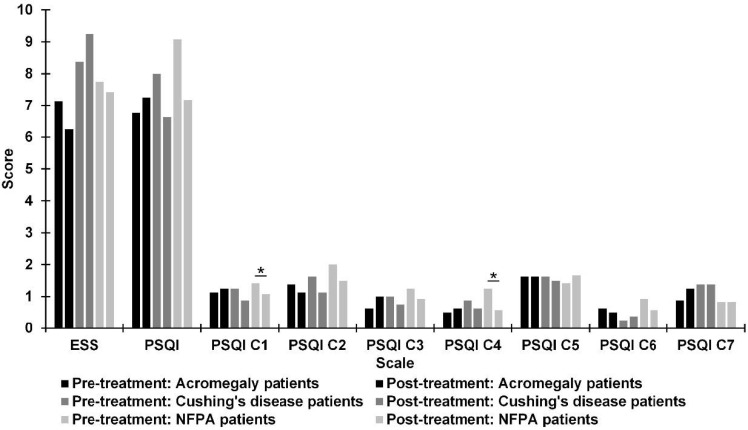
Differences between pre- and post-treatment in patients with acromegaly, patients with Cushing’s disease, and non-functioning pituitary adenoma (NFPA) patients on Epworth Sleepiness Scale (ESS) scores and Pittsburgh Sleep Quality Index (PSQI) scores. PSQI: C1 = Subjective Sleep Quality; C2 = Sleep Latency; C3 = Sleep Duration; C4 = Sleep Efficiency; C5 = Sleep Disturbances; C6 = Use of Sleeping Medication; C7 = Daytime Dysfunction. * *p* < 0.05.

Figure 2 shows the mean scores for quality of sleep from the ESS and PSQI (general scale and different subscales), preoperatively and postoperatively, for three subgroups: acromegaly, Cushing’s disease and NFPA patients. There was a significant improvement with medium effect size in Subjective Sleep Quality (*Z* = −2.00; *p* = 0.046; *r* = 0.41) and Sleep Efficiency (*Z* = −2.06; *p* = 0.024; *r* = 0.42) subscales of the PSQI only in NFPA patients.

**Figure 3:**
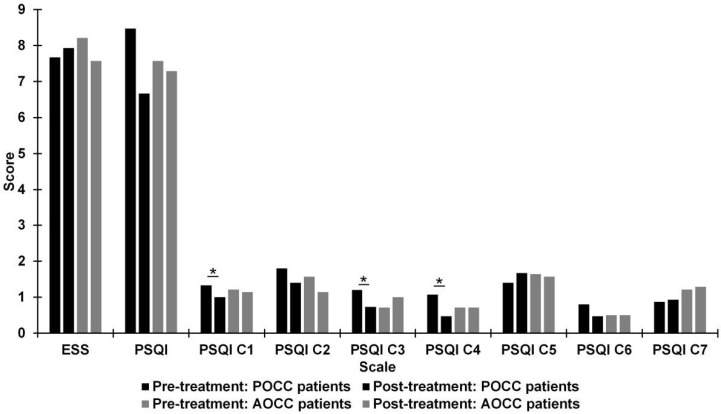
Differences between pre- and post-treatment in patients with presence of optic chiasm compression (POCC) and patients with absence of optic chiasm compression (AOCC) on Epworth Sleepiness Scale (ESS) scores and Pittsburgh Sleep Quality Index (PSQI) scores. PSQI: C1 = Subjective Sleep Quality; C2 = Sleep Latency; C3 = Sleep Duration; C4 = Sleep Efficiency; C5 = Sleep Disturbances; C6 = Use of Sleeping Medication; C7 = Daytime Dysfunction. * *p* < 0.05.

Figure 3 shows the mean scores for quality of sleep from the ESS and PSQI (general scales and different subscales), preoperatively and postoperatively, for two subgroups: patients with POCC and patients with absence of optic chiasm compression (AOCC). There was a significant improvement with medium effect size in Subjective Sleep Quality (*Z* = −2.24; *p* = 0.025; *r* = 0.41), Sleep Duration (*Z* = −2.11; *p* = 0.035; *r* = 0.38), and Sleep Efficiency (*Z* = −2.26; *p* = 0.024; *r* = 0.41) subscales of the PSQI in patients with POCC.

### 3.3. Pre- and Post-Treatment Comparison of Quality of Life

**Figure 4:**
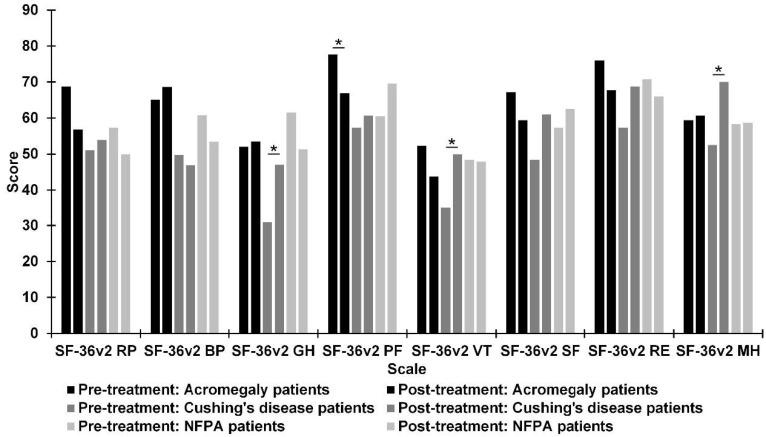
Differences between pre- and post-treatment in patients with acromegaly, patients with Cushing’s disease, and non-functioning pituitary adenoma (NFPA) patients on Short Form Health Survey version 2 (SF-36v2) scores between pre- and post-treatment. SF-36v2: BP = Bodily Pain; GH = General Health; MH = Mental Health; PF = Physical Functioning; RE = Role Emotional; RP = Role Physical; SF = Social Functioning; VT = Vitality. * *p* < 0.05.

Figure 4 shows the mean scores for quality of life from different subscales of SF-36v2, preoperatively and postoperatively, for three subgroups: acromegaly, Cushing’s disease, and NFPA patients. There was a significant deterioration with large effect size in the mean of the SF-36v2 PF subscale score (*Z* = −2.02; *p* = 0.043; *r* = 0.51) in patients with acromegaly. There was a significant improvement with large effect size in the means of scores on the GH (*Z* = −2.37; *p* = 0.018; *r* = 0.59), VT (*Z* = −2.05; *p* = 0.041; *r* = 0.52), and MH (*Z* = −2.06; *p* = 0.040; *r* = 0.52) subscales of the SF-36v2 in patients with Cushing’s disease.

**Figure 5:**
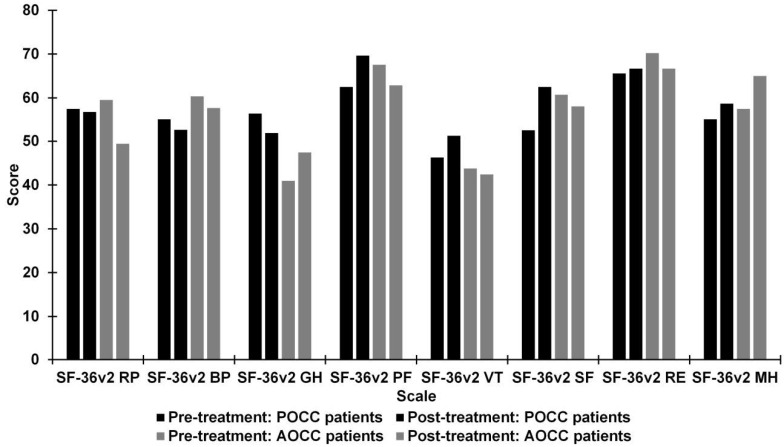
Differences between pre- and post-treatment in patients with presence optic chiasm compression (POCC) and patients with absence optic chiasm compression (AOCC) on Short-Form Health Survey version 2 (SF-36v2) scores. SF-36v2: BP = Bodily Pain; GH = General Health; MH = Mental Health; PF = Physical Functioning; RE = Role Emotional; RP = Role Physical; SF = Social Functioning; VT = Vitality.

Figure 5 shows mean scores for quality of life from different subscales of SF-36v2, preoperatively and postoperatively, for two subgroups: patients with POCC and AOCC. No significant differences were detected in the different subscales of SF-36v2 in both groups.

### 3.4. Pre-Treatment Relationship between Quality of Sleep and Quality of Life

Table 2 shows correlations between quality of sleep scores from ESS and PSQI and quality of life from SF-36v2 (different subscales) preoperatively: for three subgroups when divided by tumor type—acromegaly, Cushing’s disease, and NFPA patients; and two subgroups when divided by compression of the optic chiasm—patients with POCC and AOCC.

For patients with acromegaly, there were significant negative correlations between the PSQI global score and the following SF-36v2 subscales: BP (*R* = −0.78; *p* = 0.022), GH (*R* = −0.74; *p* = 0.035), SF (*R* = −0.78; *p* = 0.023), and RE (*R* = −0.76; *p* = 0.027). In patients with Cushing’s disease, there were significant negative correlations between PSQI global score and the GH (*R* = −0.72; *p* = 0.046) and PF (*R* = −0.75; *p* = 0.034) subscales of the SF-36v2. In patients with NFPA, there was a significant negative correlation between the ESS score and the MH subscale of the SF-36v2 (*R* = −0.66; *p* = 0.020) score and between the PSQI global score and the BP subscale of the SF-36v2 (*R* = −0.62; *p* = 0.034). In patients with POCC, there were significant correlations between the ESS score and BP (*R* = −0.53; *p* = 0.045) and MH (*R* = −0.62; *p* = 0.014) subscales of the SF-36v2. In patients with AOCC, there were significant correlations between the PSQI global score and the following SF-36v2 subscales: GH (*R* = −0.68; *p* = 0.008), PF (*R* = −0.78; *p* = 0.002), and SF (*R* = −0.62; *p* = 0.018).

### 3.5. Post-Treatment Relationship between Quality of Sleep and Quality of Life

Table 3 shows correlations between quality of sleep scores from ESS and PSQI and quality of life from SF-36v2 postoperatively: for three subgroups when divided by tumor type—acromegaly, Cushing’s disease, and NFPA patients; and two subgroups when divided by compression of the optic chiasm—patients with POCC and AOCC.

In patients with acromegaly, there was a significant correlation between the global PSQI score and the GH (*R* = −0.74; *p* = 0.038) subscale of the SF-36v2. In patients with POCC, there was a significant correlation between the global PSQI score and the RP (*R* = −0.57; *p* = 0.027) subscale of the SF-36v2. In patients with AOCC, there was a significant correlation between the global PSQI score and the MH (*R* = −0.55; *p* = 0.043) subscale of the SF-36v2.

## 4. Discussion

Patients with PA have significantly decreased QoL, which may not return to normal despite successful treatment. This study assessed the effect of microscopic TSS on QoL and sleep and also analyzed the relationships between subjective sleep quality and components of QoL.

Sleep disorders that occur in patients with pituitary adenomas have a wide variety of etiologies. Nonetheless, physical damage to the circadian rhythm system, including to the retinohypothalamic tract and/or the suprachiasmatic nucleus (SCN), may be the leading cause of sleep disorders in patients with suprasellar lesions. The retinohypothalamic tract is a monosynaptic pathway that conveys non-image-forming light impulses from intrinsically photoreceptive retinal ganglion cells, via the optic chiasm, to the SCN. The SCN, which is considered the circadian pacemaker, projects its efferents to the pineal gland, thus controlling melatonin production. Some studies have suggested that stimulation of the optic chiasm may phase-shift the SCN pacemaker in a pattern similar to that produced by light impulses [20]. Therefore, it has long been believed that optic chiasm compression by suprasellar tumors may lead to disruption of diurnal rhythms. Consistent with this view are the findings that patients previously treated for sellar tumors involving the optic chiasm, suffer from fragmented day-night activity, later habitual bedtime, later sleep onset, and shorter sleep [21,22]. Despite the evidence for persistent hypothalamic dysfunction in PA patients, Romijn did not exclude the possibility that some sleep abnormalities may be reversible after decompression of the optic chiasm [12]. Our study appears to be the first to provide evidence in this regard, as our patients with optic chiasm compression demonstrated improvement in subjective sleep quality, sleep duration, and habitual sleep performance after optic chiasm decompression by TSS, whereas no differences were observed in patients with AOCC.

It is well established that FPA patients experience circadian rhythm abnormalities as a consequence of hormonal overproduction. Sleep disturbance is a hallmark of hypercortisolism and obstructive sleep apnea syndrome, which substantially decreases QoL [23], occurs in 44 to 87.5% of patients with active acromegaly [24]. In our cohort of FPA patients, no changes in PSQI or ESS scores were observed at the time of follow-up. Similarly, Chemla et al. did not see any improvement in polysomnography parameters after successful treatment of acromegaly [25]. This could be explained by delays in the diagnosis of acromegaly and development of chronic comorbidities, which persist despite of biochemical cure. However, a recent study by Wolters et al. has shown a substantial decrease in the prevalence and severity of obstructive sleep apnea syndrome and improvement in ESS following treatment of acromegaly [26]. Importantly, the study included patients before initiation of pharmacological treatment, with a follow-up period of 2.5 years. Taking these aspects into account, the effect of TSS on QoL and sleep parameters in our cohort of acromegaly patients may not have been pronounced, as pharmacological treatment was initiated prior to the first assessment and the follow-up time was shorter.

We observed a significant improvement in subjective sleep quality and habitual sleep efficiency in NFPA patients. This was most likely an effect of optic chiasm decompression in these patients, as discussed above.

In our cohort, a significant improvement in QoL occurred only in patients with Cushing’s disease and it concerned GH, VT, and MH. It is well known that patients with Cushing’s disease experience serious mental and physical problems and, according to studies, have the lowest QoL of all PA patients [27]. Postsurgical remission rates of Cushing’s disease range from 25 to 100%, with a mean remission rate of 77.8% [28]. However, even if a biochemical cure has been achieved, QoL improves but does not normalize—even many years later [29]. Lindsay et al. evaluated QoL in Cushing’s disease patients with a mean follow-up of 3.2 years after surgery. In their study, scores for BP, VT, SF, RE, GH, and MH improved to levels similar to population norms; however, PF and RP remained significantly below norms [7]. This is in line with our study, indicating that mental rather than physical recovery is more pronounced in this group of patients. This view is supported by findings that the volume of the amygdala and the hippocampus are decreased in the active form of disease, but increase after disease remission [30], whereas muscle strength decreases even after successful treatment and remains impaired years after treatment [31]. It is worth noting that the detection of improvement may also depend on the tools used for the evaluation. For example, Santos et al. compared data obtained from 11 Cushing’s disease patients with follow-up evaluations after endocrine control of hypercortisolism. Improvement in the EuroQoL Visual Analogue Scale score was observed at 9 (±3) months, but not at 4 (±1.5) months; however, the CushingQoL score improved at 4 (±1.5) months [32].

Acromegaly affects QoL mostly through headaches, cognitive and psychopathological dysfunction, and also musculoskeletal symptoms, which usually persist after successful treatment [33]. Moreover, the delay in the diagnosis of acromegaly is raised by many authors and contributes to the occurrence of complications that worsen the QoL. In our study, despite a high remission rate of acromegaly, a surprising decline in PF occurred. The most probable explanation of this finding is the specific characteristics of the study group. Firstly, according to the Polish Society of Endocrinology, patients from the study group received somatostatin analogues (SSA) preoperatively [15], which improves QoL in patients with acromegaly [34]. Secondly, in our group of patients, recombinant human growth hormone (rhGH) was not substituted, as it was not subsidized in the country until November 2020 [35]. Valassi et al. demonstrated that in women who became GH deficient after treatment for acromegaly, QoL improved after substitution therapy with rhGH [36]. Similary to our study, van der Klaauw et al. observed progressive worsening in patients with controlled acromegaly on the PF and SF subscales [37].

We could not prove a significant change in QoL in NFPA patients following TSS, despite the fact that the effects of surgery in our group were not inferior to other studies. Results from other prospective observational studies analyzing the effects of TSS on QoL in patients with sellar masses, including NFPAs, are inconsistent, revealing very good improvements [38,39], moderate improvements [40,41,42], or no improvements [43]. Interestingly, Tanemura et al. reported that NFPA patients who required hormonal substitution scored much lower on the SF-36v2 scale postoperatively and most likely have worse prognosis for improvement [40]. Our cohort had one of the highest rates of hypopituitarism at baseline compared to other studies reporting this pathology [38,40,41,43]. A high percentage of patients receiving hormonal substitution prior to surgery was also present in the study of Wang [43], who also did not observe improvement in QoL during the 4 months of follow-up. Moreover, in a recent cross-sectional study, Vega-Beyhart et al. did not identify differences in QoL between NFPA patients with and without surgery [44]. This aspect requires further study, as identifying prognostic factors for improvement would be of great clinical significance.

Finally, in our study we have demonstrated multiple associations between subjective sleep parameters and QoL. Before surgery, these correlations differed between groups of patients divided by tumor type, which is most likely due to the various etiologies of sleep disturbances in endocrinopathies. Most correlations were observed in acromegaly patients, likely due to headaches, musculoskeletal pain, and obstructive sleep apnea, which constitute the greatest burdens of acromegaly. In patients with POCC prior to surgery, increased daytime sleepiness was associated with greater pain and poorer mental health, possibly representing the effect of the tumor mass, including headaches and disruption of the circadian pace-maker. In patients with AOCC, PSQI negatively correlated with GH, PF, and SF scores, most likely being an effect of hormonal disturbances as this group consisted mostly of acromegaly and Cushing’s disease patients. After TSS correlations between subjective sleep parameters and QoL persisted in patients with acromegaly, POCC and AOCC.

Our study has certain limitations. Firstly, conclusions from our observations must be drawn carefully due to the small study groups. This aspect is, however, difficult to overcome in single-center studies analyzing rare diseases such as Cushing’s disease or acromegaly. Secondly, there is some heterogeneity among patients with POCC, as the group consists of FPA and NFPA patients. Nevertheless, considering that in FPA patients no changes in PSQI or ESS scores were observed at follow-up, this aspect most likely does not influence interpretation of the results. Thirdly, as subjects from this study did not receive substitution therapy with rhGH, due to socioeconomic limitations, some of the observed aspects may not apply to other PA patient groups.

There are certain strengths of this study. Firstly, this was a prospective study analyzing the influence of TSS on QoL and sleep quality in the perioperative period depending on tumor type and POCC, with scarce literature in this field and discrepancies among the previously reported results. In our study group, high remission rate of FPAs and gross total resections of NFPAs was achieved, thus enabling a reliable assessment of the impact of successful TSS on QoL. The group was homogenous due to a single neurosurgeon performing TSS. Patients remained under the control of different endocrinologists in the outpatient setting, thus lowering the risk of bias while answering the questionnaires. Additionally, studies in Central Europe on QoL in PA patients have not been published and our investigation broadens knowledge in this field.

## 5. Conclusions

In conclusion, our study provided evidence that despite successful surgical treatment, most patients do not observe significant improvement in QoL during the first months after TSS, and that improvement depends on tumor type. Secondly, as far as the authors know, this is the first study to suggest that some circadian rhythm disturbances may resolve promptly after decompression of the optic chiasm. Moreover, this work supports the view that many dimensions of QoL in PA patients are associated with quality of sleep, thus implying that developing strategies to improve sleep quality may increase PA patients’ overall well-being and everyday functioning.

## Figures and Tables

**Table 1:** Baseline clinical characteristics of patients.

FPA: *n* (%)	17 (59%)
Acromegaly: *n* (%)	8 (28%)
Cushing’s disease: *n* (%)	8 (28%)
Gonadotropinoma: *n* (%)	1 (3.4%)
NFPA: *n* (%)	12 (41%)
Hypopituitarism: *n* (%)	18 (62%)
Macroadenoma: *n* (%)	26 (90%)
Below < 4 cm: *n*	24
Above > 4 cm (Giant adenoma): *n*	2
Microadenoma: *n* (%)	3 (10%)
Suprasellar tumors with optic chiasm compression (grading according to the Hardy classification system): *n* (%)	15 (52%)
Grade A	6 (21%)
Grade B	5 (17%)
Grade C	2 (7%)
Grade D	2 (7%)
Knosp scale: *n* (%)	
Grade 0	2 (6.9%)
Grade 1	4 (20%)
Grade 2	10 (34%)
Grade 3	8 (28%)
Grade 4	5 (17%)
Age: *M* (*SD*)	52.7 (15.8)
Sex	
Male: *n* (%)	15 (52%)
Female: *n* (%)	14 (48%)

FPA = functional pituitary adenoma; NFPA = non-functioning pituitary adenoma.

**Table 2:** Relationship between Epworth Sleepiness Scale (ESS) scores, Pittsburgh Sleep Quality Index (PSQI) scores, and Short-Form Health Survey version 2 (SF-36v2) scores in patients with acromegaly, patients with Cushing’s disease, patients with non-functioning pituitary adenomas (NFPA), patients with presence of optic chiasm compression (POCC), and patients with absence optic chiasm compression (AOCC) in pre-treatment.

	SF-36v2 RP	SF-36v2 BP	SF-36v2 GH	SF-36v2 PF	SF-36v2 VT	SF-36v2 SF	SF-36v2 RE	SF-36v2 MH
Patients with acromegaly								
ESS	0.28	0.14	−0.12	0.21	−0.49	−0.14	−0.25	−0.31
PSQI	−0.35	−0.78 *	−0.74 *	−0.57	−0.34	−0.78 *	−0.76 *	−0.30
Patients with Cushing’s disease								
ESS	0.14	−0.59	−0.16	−0.09	−0.11	−0.60	−0.39	−0.56
PSQI	0.59	0.10	−0.72 *	−0.75 *	0.33	−0.39	0.08	0.09
Patients with non-functioning pituitary adenomas (NFPA)								
ESS	−0.10	−0.32	−0.20	−0.16	−0.47	−0.27	−0.09	−0.66 *
PSQI	−0.37	−0.62 *	0.45	0.00	−0.11	−0.35	−0.29	−0.05
Patients with presence of optic chiasm compression (POCC)								
ESS	−0.19	−0.53 *	−0.33	0.17	−0.46	−0.41	−0.30	−0.62 *
PSQI	−0.28	0.50	0.49	−0.16	−0.03	−0.34	−0.25	0.15
Patients with absence of optic chiasm compression (AOCC)								
ESS	0.21	−0.14	−0.14	−0.06	−0.20	−0.09	−0.02	−0.45
PSQI	0.01	−0.42	−0.68 *	−0.78 *	0.09	−0.62 *	−0.45	−0.31

SF-36v2: BP = Bodily Pain; GH = General Health; MH = Mental Health; PF = Physical Functioning; RE = Role Emotional; RP = Role Physical; SF = Social Functioning; VT = Vitality. * *p* < 0.05.

**Table 3:** Relationship between Epworth Sleepiness Scale (ESS) scores, Pittsburgh Sleep Quality Index (PSQI) scores, and Short-Form Health Survey version 2 (SF-36v2) scores in patients with acromegaly, patients with Cushing’s disease, patients with non-functioning pituitary adenomas (NFPA), patients with presence optic chiasm compression (POCC), and patients with absence of optic chiasm compression (AOCC) in post-treatment.

	SF-36v2 RP	SF-36v2 BP	SF-36v2 GH	SF-36v2 PF	SF-36v2 VT	SF-36v2 SF	SF-36v2 RE	SF-36v2 MH
Patients with acromegaly								
ESS	0.07	−0.05	−0.37	0.04	−0.36	−0.09	0.01	−0.46
PSQI	−0.24	−0.02	−0.74 *	−0.18	−0.30	−0.35	−0.55	−0.66
Patients with Cushing’s disease								
ESS	−0.35	−0.70	−0.04	−0.15	0.14	0.16	−0.11	0.33
PSQI	−0.21	−0.27	0.66	−0.35	−0.01	−0.30	−0.16	0.12
Patients with non-functioning pituitary adenomas (NFPA)								
ESS	0.20	−0.25	−0.32	0.31	0.13	0.12	0.14	0.03
PSQI	−0.21	−0.31	−0.18	−0.32	−0.11	−0.15	−0.18	−0.01
Patients with presence of optic chiasm compression (POCC)								
ESS	0.12	−0.36	−0.15	0.25	−0.23	−0.23	0.01	−0.40
PSQI	−0.57 *	−0.20	−0.02	−0.49	−0.12	−0.15	−0.45	0.05
Patients with absence of optic chiasm compression (AOCC)								
ESS	0.05	−0.44	−0.24	−0.01	0.08	0.24	0.09	0.09
PSQI	−0.03	−0.10	−0.43	−0.21	−0.13	−0.16	−0.14	−0.55 *

SF-36v2: BP = Bodily Pain; GH = General Health; MH = Mental Health; PF = Physical Functioning; RE = Role Emotional; RP = Role Physical; SF = Social Functioning; VT = Vitality. * *p* < 0.05.

## Data Availability

The datasets generated during and/or analyzed during the current study are available from the corresponding author on reasonable request.
